# Identification of Major QTLs Associated With First Pod Height and Candidate Gene Mining in Soybean

**DOI:** 10.3389/fpls.2018.01280

**Published:** 2018-09-19

**Authors:** Hongwei Jiang, Yingying Li, Hongtao Qin, Yongliang Li, Huidong Qi, Candong Li, Nannan Wang, Ruichao Li, Yuanyuan Zhao, Shiyu Huang, Jingyao Yu, Xinyu Wang, Rongsheng Zhu, Chunyan Liu, Zhenbang Hu, Zhaoming Qi, Dawei Xin, Xiaoxia Wu, Qingshan Chen

**Affiliations:** ^1^College of Agriculture, Northeast Agricultural University, Harbin, China; ^2^Jilin Academy of Agricultural Sciences, Soybean Research Institute, Changchun, China; ^3^Heilongjiang Academy of Agricultural Sciences, Jiamusi Branch Institute, Jiamusi, China

**Keywords:** soybean, first pod height, QTL mapping, candidate genes, mechanized harvest

## Abstract

First pod height (FPH) is a quantitative trait in soybean [*Glycine max* (L.) Merr.] that affects mechanized harvesting. A compatible combination of the FPH and the mechanized harvester is required to ensure that the soybean is efficiently harvested. In this study, 147 recombinant inbred lines, which were derived from a cross between ‘Dongnong594’ and ‘Charleston’ over 8 years, were used to identify the major quantitative trait loci (QTLs) associated with FPH. Using a composite interval mapping method with WinQTLCart (version 2.5), 11 major QTLs were identified. They were distributed on five soybean chromosomes, and 90 pairs of QTLs showed significant epistatic associates with FPH. Of these, 3 were main QTL × main QTL interactions, and 12 were main QTL × non-main QTL interactions. A KEGG gene annotation of the 11 major QTL intervals revealed 8 candidate genes related to plant growth, appearing in the pathways K14486 (auxin response factor 9), K14498 (serine/threonine-protein kinase), and K13946 (transmembrane amino acid transporter family protein), and 7 candidate genes had high expression levels in the soybean stems. These results will aid in building a foundation for the fine mapping of the QTLs related to FPH and marker-assisted selection for breeding in soybean.

## Introduction

Soybean [*Glycine max* (L.) Merr.], which is widely grown worldwide, is a main plant oil source and food crop (Li et al., 2017). Traditional soybean cultivation depends on mechanized harvesting, which has restricted its production, and the breeding of suitable mechanized harvest-optimized varieties has become an urgent requirement for breeders (Paixao et al., 2016). First pod height (FPH) is a crucial characteristic of suitable mechanized harvesting varieties. Cultivars with a low FPH may be cut, damaged, or not harvested during mechanical harvesting (Milan et al., 2005). The main effects of planting distance, genotype, and seeding date are significant for FPH (Kang et al., 2017). Additionally, a QTL mapping analysis was applied in soybean, which laid an important foundation for cultivating new varieties suitable for mechanization (Fan et al., 2012). The FPH characteristics influence mechanized harvesting. Milan et al. (2005) investigated a genetic model of high-yield pods and concluded that the genes controlling FPH were recessive. Singh et al. (2013) hypothesized that FPH plays an important role in the economical harvesting of Faba bean. The FPH characteristics in other beans are excellent references for soybean, and the soybean FPH, comparatively, has a lower genetic coefficient of variation and higher heritability and genetic progress. FPH is a quantitative trait in soybean that is controlled by multiple factors, and is positively correlated with the yield.

Quantitative trait loci (QTL) mapping is a standard method for examining the quantitative traits. There are several methods used for QTL mapping, including interval mapping (IM; Korol et al., 1995), composite IM (CIM; Utz and Melchinger, 1996), and multiple IM (Kao et al., 1999). The QTL mapping methods have been used to examine the quantitative traits of many crops, including 100-kernel weight in maize (Raihan et al., 2016), the carotenoids content in potato (Campbell et al., 2014), the ratio of deep rooting in rice (Horii et al., 2006; Lou et al., 2015), and the seed coat surface in soybean (Otobe et al., 2015). The FPH is an important element of yield and has negative correlations with plant density and nitrogen rate in soybean breeding (Mehmet, 2008). Row spacing has no measurable effect on FPH (Grichar, 2007).

The QTLs with epistatic effects play critical roles in quantitative trait mapping (Cockerham and Zeng, 1996; Xing et al., 2002; Zhuang et al., 2002; Zhang et al., 2008). Previous studies of soybean yield and the related agronomic traits were mainly focused on the mapping of QTLs and the interactions among segregants, while paying less attention to the epistatic effects of QTLs, which are influenced by the genetic analysis and analysis limitations. Interactions between the QTLs are meaningful and control large effects (Lark et al., 1995). Wang et al. (1999) used a computer software program (QTL Mapper, version 1.0, Zhejiang University) to investigate the interval mapping of QTLs with additive, and additive × additive epistatic effects. QTL IciMapping is a commonly used software for mapping the QTLs in population (Meng et al., 2015). In the current research, we analyzed the QTLs of a recombinant inbred line (RIL) population using the CIM and ICIM methods, and detected the QTLs with epistatic effects.

Although soybean FPH has been genetically analyzed in a few preliminary studies, the main effects of soybean QTLs associated with the FPH have not been reported over many years. Furthermore, only a few candidate genes for soybean FPH have been reported. In this study, a 147 RIL population was used with a previously constructed high-density genetic map (Qi et al., 2014). The population was planted over 8 consecutive years for phenotypic observations to identify the main-effect QTLs. Epistatic effects were also analyzed to better understand the main-effect QTLs’ interactions. Furthermore, the candidate genes of soybean FPH were annotated, and qRT-PCR validation was conducted. Our results provide new QTLs associated with soybean FPH, which may improve soybean high-yield breeding and the selection of soybean varieties adapted to mechanized harvesting.

## Materials and Methods

### The FPH in an RIL Population Over 8 Years

The population consisting of 147 RILs was derived from a cross between two ILs, the America cultivar Charleston (♀) and the high protein cultivar Dongnong594 (♂). There were significant differences in FPH-associated traits between parents, and more significant differences in FPH-associated traits among the RILs. In November of each year, the parental materials were selected and 147 RILs, of 5 strains, with generally consistent growing trends were observed. Phenotypic measurements of the FPH were repeated three times during field harvesting. The FPH was recorded in cm from the ground to the bottom of the first pod over the cotyledon node (Grichar, 2007). The total length of the high-density genetic soybean map was 2,655.68 cm and included 5,308 specific-length amplified fragment markers, covering 20 linkage groups (LGs), with an average distance of 0.5 cm between the adjacent markers (Qi et al., 2014).

### Planting Conditions and Field Management

From 2006 to 2015, except for 2011 and 2012, soybean was planted in the research field of the Northeast Agricultural University, Harbin, Heilongjiang Province (Harbin, latitude 45°74′N, longitude 126°72′E). A random block design with three replicates was used over the 8 years. The row length was 5 m, where 80 seeds were sown per row, and the fields were under general management. A one-way analysis of variance (ANOVA) and least significance difference test (LSD) multiple comparison of soybean FPH over 8 years were conducted using SPASS 17.0. Based on a variance analysis, the AOV model in QTL IciMapping version 3.2 was used to determine the broad-sense heritability (h^2^):

$$\begin{array}{c} h^{2}{=r\delta g}^{2}/\left(\delta e^{2}{+r\delta g}^{2}\right) \end{array}$$

where r indicates the number of replications; δg^2^ indicates the genetic variance component; and δg^2^ indicates the environmental variance component.

### QTL Analysis of Soybean FPH in the RIL Population

Windows QTL Cartographer version 2.5 was used to analyze the QTLs using a CIM model. Phenotypic data were analyzed using the 1,000 permutation test, and the significance was set at the *P* = 0.05 level to detect QTL. QTL IciMapping version 3.2 was used for the epistatic effect analysis. Mapping software MapChart version 2.2 was used for constructing the linkage map. The QTL naming was based on the method of McCouch (1997). Briefly, the QTL name was constructed as follows: q + trait name + LG or LG number + “-” + QTL number. The QTLs detected by two methods were used for QTL integration. The chromosome segment substitution line (CSSL) population, produced by the cross between the Chinese cultivar SN14 and wild soybean (*Glycine soja* Sieb. and Zucc.) line ZYD00006, was used for verifying the consensus QTLs. The genetic map was constructed by Xin et al. (2016). The field management of the CSSL population was the same as used for the RIL plants.

### Gene Prediction of Major QTL Intervals

The ‘Williams 82.a2.v1’ genome has enough common genetic information to be defined as the reference genome for predicting the candidate genes. Soybase^1^ and the previously constructed high-density genetic map provided the genetic distances and physical positions that were used to determine the physical positions of the major QTL intervals. Using the genomic information provided by Phytozome^2^, and referring to the ‘Williams 82.a2.v1’ genome sequence, the soybean genes in the major QTL intervals were identified and the corresponding gene annotations were performed. Candidate genes involved in the growth and development of soybean were selected from the gene annotation data, which came mainly from the databases listed in **Supplementary Table S1**.

### RT-qPCR Analysis of Soybean FPH Candidate Genes

Five materials were selected, where two (RIL-86 and RIL-101) of the materials were high FPH materials and three others (RIL-2, RIL-112, and RIL-141) were low FPH materials. Lines were chosen that had significant phenotypic differences between the high-value materials and low-value materials at *P* = 0.05 levels (**Supplementary Table S2**). Tissues from the stem apices of these five lines were collected at the ternate compound-leaf stage and stored at -80°C for RNA isolation. Experiments were performed by collecting three independent groups of samples, where each replicate was a pooled sample of three individual plants, and three technical repetitions were performed simultaneously.

Total RNA was extracted from plant materials using TRIzol reagent (Invitrogen^3^) according to the manufacturer’s instructions. cDNA was synthesized using Prime Script TMRT and a reagent reverse transcription kit, *GmUKN1* (Genbank ID: 103870138), was used as an internal control. Real-time quantitative PCR (RT-qPCR) was performed using SYBR qPCR Mix (TransGen) on a LightCycler 480 System (Roche). The RT-qPCR reaction conditions were as follows: pre-denaturation cycle at 95°C for 30 s; followed by 40 cycles of 95°C for 5 s and 60°C for 30 s. The relative expression of FPH candidate genes from soybean stems was calculated using the following:

$$\begin{array}{c} 2\left(\Delta C_{t}\right)\left[\Delta C_{t}{=C}_{t}\left(GmUKN1\right){-C}_{t}\left(\mathrm{target}\mathrm{genes}\right)\right] \end{array}$$

The ‘Williams 82’ gene sequences’ 3’UTR-ends from the Phytozome website^2^ were used with Primer Premier 5.0 to design specific primers (**Supplementary Table S3**).

## Results

### FPH Phenotypic Information Over 8 Years

**FIGURE 1:**
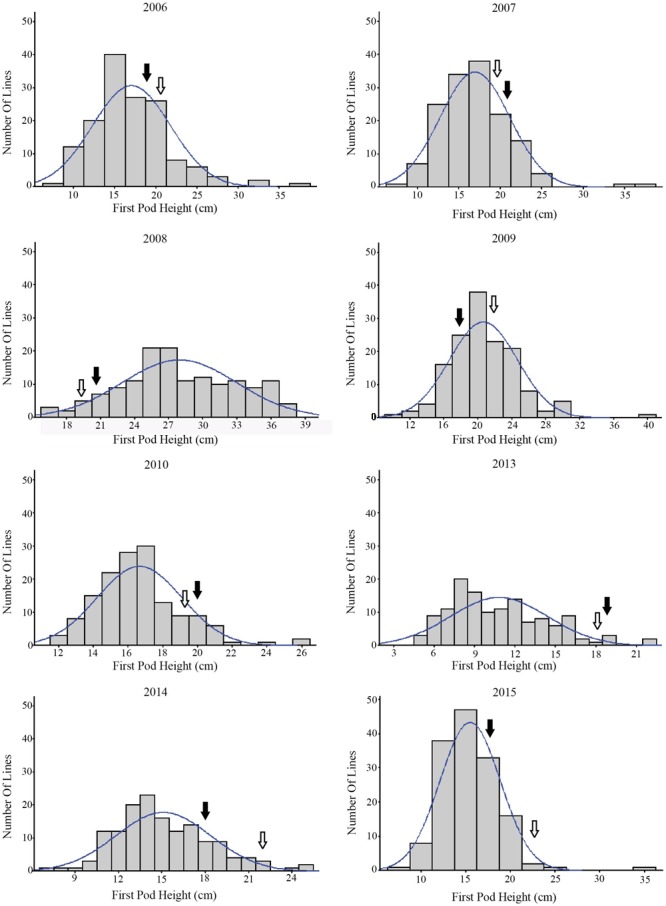
The frequency distribution of the soybean FPH in the RIL population over 8 years.

The phenotypic information of RILs from 2006 to 2015 (except 2011 and 2012) are shown in **Table 1**. Over the 8 years, the average FPH of ‘Charleston’ was 2.21 cm less than that of ‘Dongnong594,’ except in 2007, 2008, and 2013. The FPH of the RIL population was significantly different in every year, ranging from 5.21 to 39.21 cm. In 2008, the phenotypic information had a maximum standard deviation (SD) and a coefficient of variation (CV) of 5.59 and 31.22%, respectively. The minimum SD and CV occurred in 2010, at 2.46 and 6.03%, respectively. The maximum and minimum kurtosis were 15.55 in 2006 and 0.08 in 2008, respectively. The maximum and minimum skewness were 2.75 in 2006 and 0.34 in 2013, respectively. The traits revealed large quantitative variability levels and fairly normal frequency distributions in different years (**Figure 1**). The RILs showed a general shift in distributions toward lower trait values. Thus, this population was suitable for QTL mapping analysis.

**Table 1:** The soybean FPH per parental cultivar and RIL population plants from 2006 to 2015 (except 2011 and 2012).

Parent			Population						
Year/site^a^	Mean Charleston(cm) (female parent)	Mean Dongnong594(cm) (male parent)	Max^b^ (cm)	Min^c^ (cm)	Mean^d^ (cm)	SD^e^	CV^f^ (%)	Kurtosis^g^	Skewness^h^
2006	20.80	19.50	38.30	7.40	17.00	5.49	30.16	15.55	2.75
2007	19.30	20.90	37.30	8.20	16.91	4.22	17.82	4.23	1.30
2008	20.20	20.80	37.25	16.47	27.92	5.59	31.22	0.08	0.44
2009	21.40	18.30	39.21	10.87	20.62	4.67	21.80	11.18	2.20
2010	19.80	19.70	26.37	11.60	16.64	2.46	6.03	2.29	0.99
2013	19.60	19.70	22.20	5.21	10.79	3.95	15.62	0.20	0.34
2014	22.30	18.47	24.97	7.13	15.07	3.29	10.82	0.25	0.53
2015	21.48	18.75	35.75	7.54	15.45	3.38	11.45	7.53	1.59

*^*a*^Site: all trials were sown at one SITE (HRB). ^*b*^Max (cm): the maximum of the phenotypic data from the RILs. ^*c*^Min (cm): the minimum of the phenotypic data from the RILs. ^*d*^Mean (cm): the average values of the phenotypic data from the RILs. ^*e*^SD: standard deviation of the phenotypic trait. ^*f*^CV (%): coefficient of variation of the phenotypic trait. ^*g*^Skewness: skewness of the phenotypic trait. ^*h*^Kurtosis: kurtosis of the phenotypic trait.*

### Environmental Conditions and Multiple Comparisons Analysis

The meteorological data over 8 years in Harbin are shown in **Supplementary Tables S4–S6**. The minimum and maximum annual average precipitation levels were 415.8 mm in 2014 and 633.5 mm in 2013, respectively (**Supplementary Table S4**). The minimum mean temperature in Harbin was 4.3°C in 2013, and the maximum mean temperature was 6.6°C in 2007 and 2008 (**Supplementary Table S5**). The maximum sunshine period was 2,379.2 h, in 2007, and the minimum sunshine period was 2,023.5 h in 2013 (**Supplementary Table S6**). The mean differences were significant at the 0.05 level, and the mean FPH values in 2008, 2009, and 2013 were significantly different from the mean FPH values in other years. Additionally, the mean FPH data in 2006 and 2007 were not significantly different from that of 2010, and the mean FPH data from 2006 was not significantly different from that of 2007. The mean FPH data from 2014 was not significantly different from that of 2015 (**Supplementary Table S7**). The h2 from 2006 to 2015 is 0.797, 0.778, 0857,0.800, 0.799, 0.897, 0.823, and 0.833, respectively (**Table 2**), and the overall broad heritability across 8 years is 0.160. The broad heritability per year is higher than the whole. The results also show that the FPH is greatly affected by the environmental factors.

**Table 2:** The broad-sense heritability for soybean FPH.

Item	2006	2007	2008	2009	2010	2013	2014	2015	Total
**SS^a^**	0.426	2.263	85.770	81.108	13.303	6.512	5.484	2.489	197.043
**MS^b^**	0.213	1.132	42.885	40.554	6.652	3.256	2.742	1.244	12.315
**LSD(0.05)**	3.730	3.460	3.233	3.105	1.900	2.151	2.366	2.354	5.940
**LSD(0.01)**	4.914	4.558	4.260	4.091	2.503	2.834	3.117	3.101	7.808
**h^2^**	0.797	0.778	0.857	0.800	0.799	0.897	0.823	0.833	0.160

*^*a*^SS: sum of squares. ^*b*^MS: mean square.*

### QTL Mapping Analysis for FPH in the RIL Population

The FPH data were analyzed using the CIM method of Windows QTL Cartographer version 2.5. The results of the QTL analysis are presented in **Table 3**.

**Table 3:** QTL mapping of soybean FPH using the CIM method over 8 years.

Environment^a^	QTL	Linkage group	LOD^b^	*R*^2^ (%)^c^	ADD^d^	Start position (Mb)	End position (Mb)
2006	*qFPH-d2-1*	D2	3.74	9.20	–1.77	14.70	14.71
2007	*qFPH-l-3*	L	3.99	10.40	–1.40	19.22	20.01
2008	*qFPH-m-1*	M	3.85	9.90	–2.18	15.87	18.41
2009	*qFPH-d2-2*	D2	3.79	8.50	–2.64	26.57	27.82
2009	*qFPH-d2-3*	D2	6.17	14.10	3.94	18.48	19.44
2013	*qFPH-j-1*	J	5.57	15.30	–2.02	25.34	31.71
2014	*qFPH-d1b-1*	D1b	5.18	12.70	1.28	49.12	49.98
2014	*qFPH-d1b-4*	D1b	3.95	9.40	–1.08	36.05	36.81
2015	*qFPH-d1b-2*	D1b	4.00	9.70	1.20	48.82	49.98
2015	*qFPH-d1b-3*	D1b	4.70	12.10	–1.29	39.32	41.72
2015	*qFPH-i-1*	I	2.65	6.50	1.07	45.55	45.74

*^*a*^Environment: all trials were sown at one environment (HRB). ^*b*^LOD: log of odds. ^*c*^*R*^2^ (%): the contribution rate of the QTL. ^*d*^ADD: the additive effects contributed by the QTL.*

In total, 11 QTLs associated with the FPH were detected during 2006 to 2015 (except 2011 and 2012). Four, three, one, one and one QTLs were located on LGs D1b, D2, L, M and J, respectively. The R^2^ (the contribution rate of the QTL) for all of the QTLs ranged from 6.50 to 15.30%, with the log of odds (LOD) values between 2.65 and 6.17. *qPH-j-1* had the maximum R^2^ of 15.30% with an LOD value of 5.57, while *qPH-i-1* had the minimum R^2^ of 6.5% with an LOD value of 2.65. On LG D2, *qPH-d2-1*, which had the minimum map distance of 0.001 Mb, was mapped between 14.70 and 14.71 Mb, thereby explaining ∼9.20% of the variation and having an additive effect of -1.77. On LGM, *qPH-m-1*, which had the maximum map distance of 2.54 Mb, was mapped between 15.87 and 18.41 Mb, thereby explaining ∼9.90% of the variation and having an additive effect of -2.18.

The major QTLs, *qPH-d1b-1* and *qPH-d1b-2*, were detected in both 2014 and 2015 at the same time and were distributed on LG D1b. Their LOD scores were 5.18 and 4.00, respectively, and their *R*^2^ values were 12.70 and 9.70, respectively. They had additive effects of 1.28 and 1.20, respectively, and physical distances of 0.86 and 1.16 Mb, respectively.

### QTL Analysis With Genetic Main and Epistatic Effects of the FPH in the RIL

**FIGURE 2:**
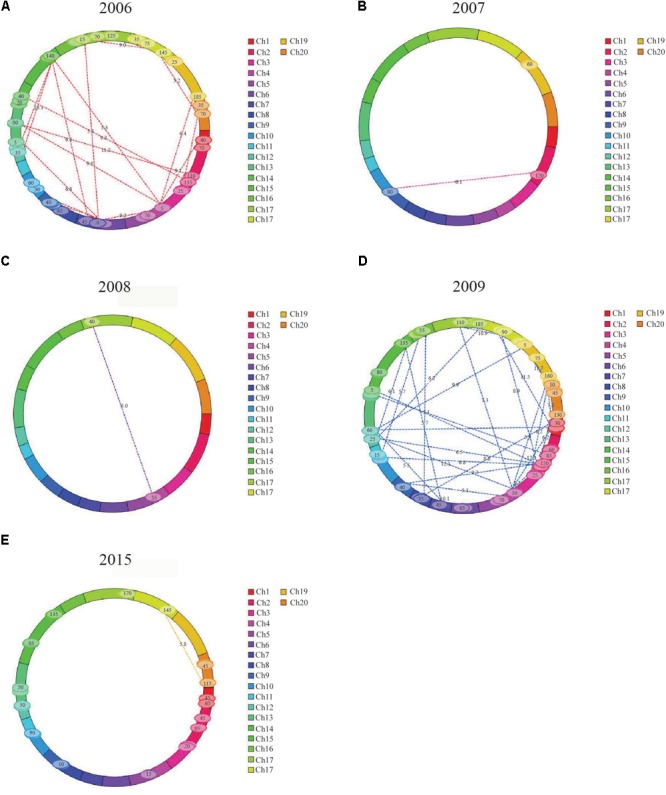
Pairs of QTLs showing significant additive × additive epistatic effects for FPH in the RIL population. **(A)** Thirty-two pairs of QTLs showing significant additive × additive epistatic effects for FPH in 2006. **(B)** One pair of QTLs showing significant additive × additive epistatic effects for FPH in 2007. **(C)** One pair of QTLs showing significant additive × additive epistatic effects for FPH in 2008. **(D)** Forty-two pairs of QTLs showing significant additive × additive epistatic effects for FPH in 2009. **(E)** Eleven pairs of QTLs showing significant additive × additive epistatic effects for FPH in 2015.

The IciMapping method was used to analyze the epistatic effects of FPH over 8 years, and 90 QTL pairs showed epistatic effects (**Table 4, Figure 2**, and **Supplementary Table S8**).

**Table 4:** Summary of the epistatic effect analysis of soybean FPH.

Environment^a^	Pairs number of Epistatic effects	LOD	PVE (%)	ADD1^b^	ADD2^c^	ADD by ADD^d^
2006	32	5.10–13.57	0.73–1.65	–10.44–9.88	–10.37–10.04	–10.60–4.05
2007	2	5.17–6.07	9.49–11.93	4.16–4.93	–4.39–3.73	–5.82–4.84
2008	1	5.01	16.66	1.40	–1.64	–2.86
2009	42	5.09–12.57	0.61–1.40	–6.77–7.48	–6.99–6.76	–7.25–2.51
2015	13	5.01–6.76	1.49–2.47	–5.37–5.47	–5.46–5.63	–5.42–2.39

*^*a*^Environment: all trials were sown at one environment (HRB). ^*b*^ADD1: the additive effects contributed by the first QTL loci. ^*c*^ADD2: the additive effects contributed by another QTL loci. ^*d*^ADD by ADD: the additive effects contributed by two QTL loci.*

In 2006, 32 pairs of loci with epistatic effects were detected, and the phenotypic variation explained (PVE) ranged from 0.73 to 1.65%, with the LOD values between 5.10 and 13.57. The pair between *qPH-g-5* and *qPH-i-5* had the maximum additive × additive value of -4.05. The pair between *qPH-c1-4* and *qPH-c2-3* had the minimum additive × additive value of -10.60. There were no significant interaction effects between the epistasis and the environment.

In 2007, two pairs of loci with epistatic effects were detected, and the PVE ranged from 9.49 to 11.93%, with the LOD values between 5.17 and 6.07. The pair between *qPH-d1b-6* and *qPH-k-6* had the maximum additive × additive value of -4.84, and the pair between *qPH-l-4* and *qPH-l-5* had the minimum additive × additive value of -5.82. There were no significant interaction effects between the epistasis and the environment.

In 2008, one pair of loci with epistatic effects were detected, and the PVE was 16.66, with an LOD value of 5.01. The pair between *qPH-c1-3* and *qPH-d2-2* had an additive × additive value of -2.86. There was no significant interaction effect between the epistasis and the environment.

In 2009, 42 pairs of loci with epistatic effects were detected, and the PVE ranged from 0.61 to 1.40%, with the LOD values between 5.09 and 12.07. The pair between *qPH-m-4* and *qPH-h-6* had the maximum additive × additive value of -2.51. The pair between *qPH-d1b-2* and *qPH-d1b-3* had the minimum additive × additive value of -7.25. There were no significant interaction effects between the epistasis and the environment.

In 2015, 13 pairs of loci with epistatic effects were detected, and the PVE ranged from 1.49 to 2.47%, with the LOD values between 5.01 and 6.76. The pair between *qPH-g-5* and *qPH-i-6* had the maximum additive × additive value of -2.39, and the pair between *qPH-o-3* and *qPH-o-4* had the minimum additive × additive value of -5.42. There were no significant interaction effects between the epistasis and the environment.

### Summary of the QTL and Epistatic Effect Analyses

Among the 90 pairs of loci with epistatic effects, three pairs revealed epistatic effects between the major QTLs, having PVEs of 1.07, 1.33, and 2.46%, with the LOD values of 5.45, 9.88, and 5.01, respectively. They were located on LGs M, D1b, and D2, respectively, and appeared in 2006, 2009, and 2015, respectively. In total, 12 pairs of loci revealed epistatic effects between the major QTLs and non-major QTLs, having PVEs ranging from 1.08 to 16.66%, and with the LOD values ranging from 5.01 to 9.88. They occurred in 2006, 2008, 2009, and 2015. Other pairs of loci revealed epistatic effects between the non-major QTLs, having PVEs ranging from 0.61 to 11.93%, and with the LOD values ranging from 5.09 to 13.57. They had additive × additive values ranging from -10.29 to -2.39.

### Validation of Consensus QTLs

**FIGURE 3:**
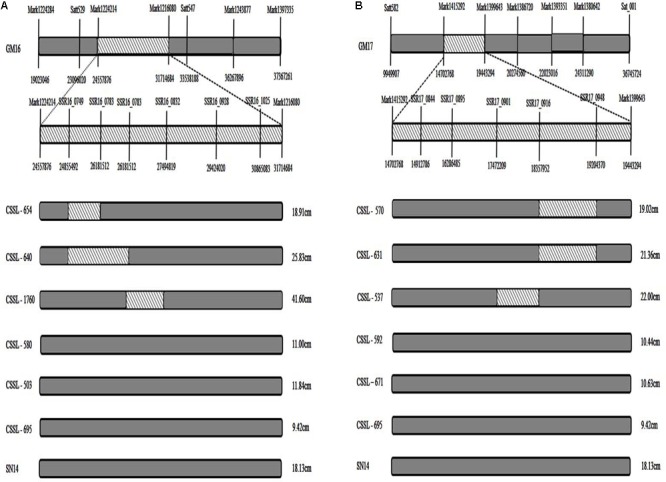
The distribution of substituted wild soybean chromosomal segments in CSSLs. **(A)** The distribution of substituted wild soybean chromosomal segments in CSSLs on GM16. **(B)** The distribution of substituted wild soybean chromosomal segments in CSSLs on GM17. Substituted wild soybean chromosomal segment.

In comparing the positions of the 11 consensus QTL intervals, four were found to be in the same region of the CSSL gene map (Xin et al., 2016). Using Soybase^1^ and the major QTL intervals of the RIL population, the corresponding markers were filtered and the fragments were screened to verify the CSSL population by polyacrylamide gel electrophoresis, and the consensus QTLs associated with FPH on GM16 (24.56–31.71 Mbp) had a corresponding region in the substituted wild soybean chromosomal segment in CSSL-654, CSSL-640, and CSSL-1760 (Satt529–Satt547) (Xin et al., 2016; **Figure 3A**). However, there was no corresponding region in the substituted wild soybean chromosomal segment in CSSL-580, CSSL-503, and CSSL-695. The phenotypic data for the FPH of CSSL-654, CSSL-640, and CSSL-1760 were greater than the corresponding data for the recurrent parent ‘SN14’ (**Figure 3A**). However, the phenotypic data for the FPH of CSSL-580, CSSL-503, and CSSL-695 were lower than the corresponding data for ‘SN14.’ Thus, the substituted wild soybean chromosomal segment had an important influence on the FPH in soybean. The other three consensus QTLs associated with the FPH on GM17 (9.95–36.74 Mb) had a corresponding region in the substituted wild soybean chromosomal segment in CSSL-570, CSSL-631, and CSSL-537 (Satt582–Sat_001) (Xin et al., 2016; **Figure 3B**). However, there was no corresponding region in the substituted wild soybean chromosomal segment in CSSL-592, CSSL-671, and CSSL-695. The phenotypic data for the FPH of CSSL-570, CSSL-631, and CSSL-537 were greater than the corresponding data for the recurrent parent ‘SN14’ (**Figure 3B**). The phenotypic data for the FPH of CSSL-592, CSSL-671, and CSSL-695 were lower than the corresponding data for ‘SN14.’ Thus, the additive effects of the consensus QTLs had different directions, which corroborated the phenotypes of the CSSLs.

### Candidate Gene Mining in Major QTL Intervals for Soybean FPH

**FIGURE 4:**
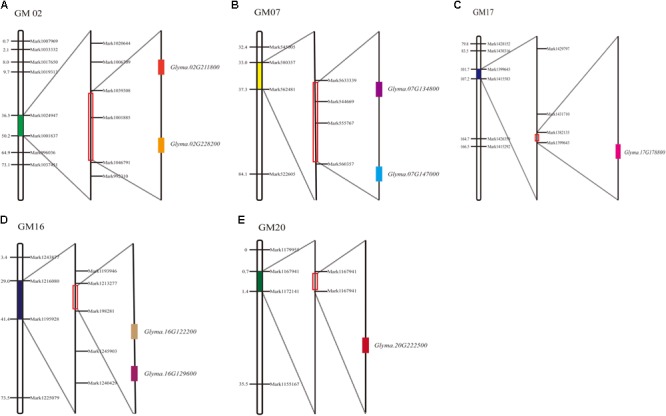
Soybean FPH-associated QTLs and candidate gene distribution. **(A)** *Glyma.02G211800* and *Glyma.02G228200* distributed in GM02. **(B)** *Glyma.07G134800* and *Glyma.07G147000* distributed in GM07. **(C)** *Glyma.17G178800* distributed in GM17. **(D)** *Glyma.16G129600* and *Glyma.16G122200* distributed in GM16. **(E)** *Glyma.20G222500* distributed in GM20.

Among the 11 major QTL intervals, 3 of them, *qFPH-d2-1, qFPH-d1b-1*, and *qFPH-d1b-*2, contained no genes. In total, 1,526 candidate genes were screened from the gene annotation data set (**Supplementary Table S9**) and 8 were involved in the soybean FPH growth pathway according to the gene ontology (GO) and annotation information (**Supplementary Table S10**). The major QTL intervals in which these related genes are located, the homologous genes in Arabidopsis, and the related GO annotations are shown (**Figure 4** and **Supplementary Tables S3, S6**). Ko04075 is involved in the plant hormone signal transduction pathway (Miao et al., 2016), which includes K14486 (auxin response factor 9), K14488 [small auxin (AUX) up RNA (SAUR) family protein], K14498 (serine/threonine-protein kinase SRK2), and K13946 (transmembrane amino acid transporter family protein). Four homologous genes were predicted in Arabidopsis and associated with the pathway. *Glyma.07G134800* occurred in the KEGG pathway (pathway_id K14486) associated with the AUX response factor, which participates in the generation of AUX/indole acetic acid and SAUR, to promote cell enlargement and plant growth. The Arabidopsis homolog of *Glyma.07G134800* is *AT4G23980*, which contains the AUX-responsive family protein domain, and participates in changing the state or activity of a cell or an organism as a result of a hormone stimuli. *Glyma.16G129600* occurred in a KEGG pathway (pathway_id K14488), and the Arabidopsis homolog of *Glyma.16G129600* is *AT4G34760*, which contains an AUX-responsive family protein domain. Lavy et al. (2016) revealed that the AUX/indole acetic acid functions and the AUX responses are related to plant growth. *Glyma.17G178800* occurred in a KEGG pathway (pathway_id K14498) that participates in carotenoid biosynthesis, and SNF1-related protein kinase (SnRK2) promotes abscisic acid-responsive element binding-factor generation and then stomatal closure and seed dormancy. The Arabidopsis homolog of *Glyma.17G178800* is *AT5G66880*, which contains a STKc_SnRK2-3 domain, and plays a role in plant tolerance to salinity and abscisic acid (Yang et al., 2012). *Glyma.07G147000* occurred in a KEGG pathway (pathway_id K13946) that participates in tryptophan metabolism, catalytic transport inhibitor responses, and ubiquitin-mediated proteolysis. The Arabidopsis homolog of *Glyma.07G147000* is *AT2G38120*. GO:0004722, which is involved in protein serine/threonine phosphatase activity, including protein serine phosphate and H_2_O-generated protein serine and phosphate, as well as protein threonine phosphate and H_2_O-generated protein threonine and phosphate. Two homologous genes were predicted in Arabidopsis and associated with the pathway. *Glyma.16G122200* and *Glyma.02G228200* occurred in this pathway, and the Arabidopsis homolog of *Glyma.16G122200* is *AT3G51370*. They contain protein phosphatase – 2Cc superfamily domains, and related research shows that the domain promotes cell expansion (Spartz et al., 2014). The Arabidopsis homolog of *Glyma.02G228200* is *AT2G30020*. They contain an AUX influx permease, PLN03074, domain. *AT2G30020* plays a role in plant hormone signal transduction pathways (Dong et al., 2015). GO:0016787 is involved in the hydrolase activity pathway, including C-O, C-N, C–C, and phosphoric anhydride bonds. The *Glyma.20G222500* gene occurs in this pathway, and the Arabidopsis homolog of *Glyma.20G222500* is *AT2G39840*, which contains a metallophosphatase–protein phosphatase type 1–kelch-like domain, which is related to continuous cell division in the meristem (Ogawa et al., 2011). The Arabidopsis homolog of *Glyma.02G211800* is *AT5G49980*. They contain the AMN1 super family domain, and *AT5G49980* participates in the AUX synthesis pathway (Prigge et al., 2016).

### Expression Analysis of Candidate Genes for Soybean FPH

**FIGURE 5:**
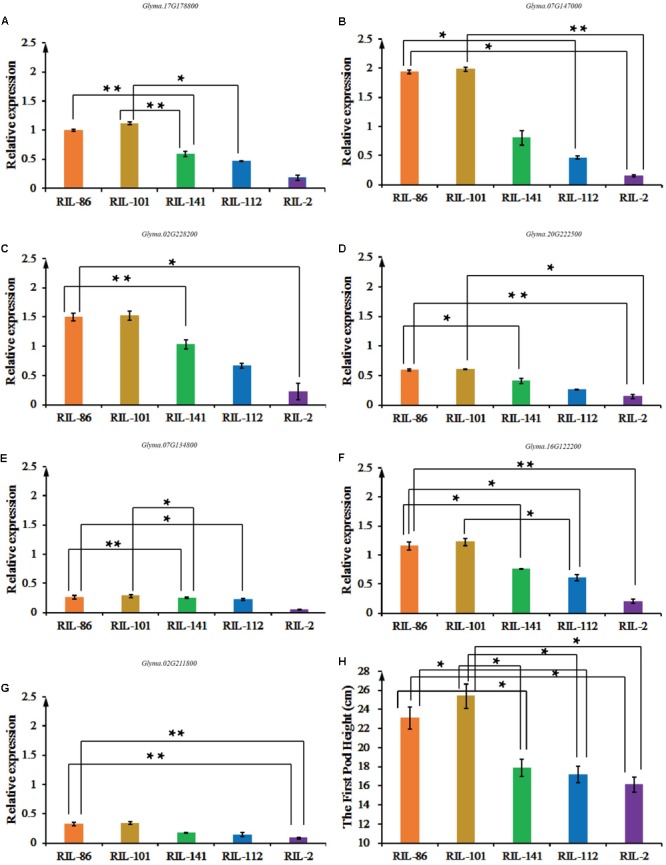
The expression levels of *Glyma.02G211800, Glyma.16G122200, Glyma.17G178800, Glyma.20G222500, Glyma.07G134800, Glyma.07G147000*, and *Glyma.02G228200* among ‘Charleston,’ ‘Dongnong594,’ RIL-2, RIL-22, RIL-75, RIL-112, and RIL-141, and the FPH-associated phenotypic data. **(A)** RT-qPCR analysis of *Glyma.02G211800* expression. **(B)** RT-qPCR analysis of *Glyma.16G122200.* **(C)** RT-qPCR analysis of *Glyma.07G134*800. **(D)** RT-qPCR analysis of *Glyma.20G222500*. **(E)** RT-qPCR analysis of *Glyma.07G134800.* **(F)** RT-qPCR analysis of *Glyma.07G147000.* **(G)** RT-qPCR analysis of *Glyma.02G228200.* Three independent groups of samples were collected, and each replicate is a pooled sample of three individual plants. Three technical repetitions were performed. **(H)** The FPH was measured in cm from the ground to the bottom of the first pod over the cotyledon node. Significant differences in comparison with the FPH of ‘Charleston’ are indicated with asterisks. ^∗^0.05 > *P* ≥ 0.01, ^∗∗^0.01 > *P* ≥ 0.001.

*Glyma.16G129600* was not expressed in soybean stems, while *Glyma.02G211800, Glyma.16G122200, Glyma.17G178800, Glyma.07G134800, Glyma.20G222500, Glyma.07G147000*, and *Glyma.02G228200* were expressed in soybean stems, suggesting that they have essential roles during the plant growth and development stage (**Figures 5A–G**). The eight-year average FPH from the five copies of the experimental materials is shown in **Figure 5H**. The FPH values of the RIL-86 and RIL-101 were greater than those of the other three experimental materials. RIL-101 had the most prominent phenotypic traits, and seven candidate genes had their greatest expression levels in RIL-101 compared with that in the other experimental materials. The experimental material of RIL-2 had the lowest FPH, and the relative expression levels of the seven candidate genes were the smallest compared with that in the other experimental materials. The variations in the seven genes’ expression levels were generally consistent with the variations in the FPH. The soybean FPH was greater in the experimental materials having greater candidate gene expression levels compared and vice versa (**Figures 5A–G**). The expression of *Glyma.07G134800* and *Glyma.17G178800* were not as obvious as those of the other six genes, and the difference in the total expression of *Glyma.07G134800* and *Glyma.17G178800* were weaker in RIL-86, RIL-101, RIL-112, and RIL-141, but the expression level was generally consistent with the variation in FPH. Thus, the qRT-PCR results indicated that the greater expression levels of the seven candidate genes may be involved in soybean growth and development, and contribute to increases in FPH during this stage.

## Discussion

Soybean FPH is a significant component of yield and plays an important role in the efficiency of mechanical harvesting. It is of great significance to find an FPH-associated QTL to help guide molecular-assisted breeding of soybean that increases the efficiency of mechanical harvesting and, thereby, soybean yield.

The agronomic traits of the RIL population showed a normal distribution, and the QTL mapping of the population will improve the mapping accuracy (Chen et al., 2005). Main-effect QTLs are important for studying the soybean agronomic traits, and they can be used for molecular-assisted breeding. At present, most researches focus on how to obtain the main-effect QTLs. In the current study, 11 major QTLs associated with soybean FPH were detected over 8 years and were located on LGs D1b, D2, L, M, and J. Additionally, 90 QTL pairs that were detected revealed additive × additive epistatic effects. Liang et al. (2014) detected two QTLs associated with the soybean FPH in an RIL population that were located on the LGs, J and M. There is an overlap for both *qFPH-j-1* and *qFPH-m-1*, which indicates the reliability of the research. To further verify the reliability of our results, CSSLs were used. Four of them (*qFPH-d2-1, qFPH-d2-2, qFPH-d2-3*, and *qFPH-j-1*) were found in the same region of the CSSL map (Xin et al., 2016). The phenotypes differ significantly between materials depending on whether these four loci from the wild soybean chromosome fragments are present separately and on the recurrent parent. The substituted wild soybean chromosomal segment has an important influence on the FPH in soybean. Thus, these QTLs are highly likely to affect the FPH. In many studies, the FPH was positively associated with plant height (Ramteke et al., 2012). In the current study, 5 of the 11 QTLs, *qFPH-d1b-3, qFPH-d1b-4, qFPH-d2-2, qFPH-l-3*, and *qFPH-m-1*, correspond to the previously reported soybean plant height QTL (Lark et al., 1995; Wang et al., 2004; Reinprecht et al., 2006; Kim et al., 2012). The increase in plant density may increase the FPH and plant height, which then increases the harvest index and seed yield (Mehmet, 2008). On the LG D1b, four QTLs, namely *qFPH-d1b-1, qFPH-d1b-4, qFPH-d1b-2*, and *qFPH-d1b-3*, were found in the near region in 2014 and 2015, indicating that this region should have pivotal genes (or gene clusters) that affect the FPH. In this region, QTLs underlying plant height and lodging were also found (Kabelka et al., 2004). As there is a correlation between plant height, FPH, and lodging, this region may contain important gene(s) that control these three traits.

Epistatic effects are the main factors that affect the soybean agronomic traits. They are an important genetic component of quantitative trait variation, being determined by the specificity of the QTL locus. Recently, several complicated traits with epistatic effects have been analyzed (Hayman, 1958; Martin et al., 2002; Xing et al., 2002). In this study, 90 pairs of epistatic-effect loci were detected. The epistatic interactions between *qFPH-m-1* and *qFPH-m-3, qFPH-m-1* and *qFPH-f-1*, and *qFPH-l-3* and *qFPH-l-2* were identified for the first time. In the region containing *qFPH-m-3, qFPH-f-1*, and *qFPH-l-2*, QTLs underlying plant high had been found in other genetic populations (Mansur et al., 1993; Lark et al., 1995; Specht et al., 2001; Gai et al., 2007; Pathan et al., 2013). Although these three QTLs were not the major main-effect QTLs, they have epistatic effects on the major main-effect QTLs. Thus, the FPH is controlled by many genes, even though some genes have small effects. However, these small-effect loci may be unstable in different environments or years. Here, there were no visible epistatic loci detected in 2010, 2013, and 2014.

Until now, there have been few reports on soybean genes associated with FPH. Based on the physical positions of the 11 major QTL intervals, 1,526 candidate genes were screened from the gene annotation data. The pathways associated with plant growth are K14486 (AUX response factor), K14488 (SAUR family protein), K06269 (serine/threonine-protein phosphatase PP1 catalytic subunit; EC:3.1.3.16), K14498 (serine/threonine-protein kinase SRK2; EC:2.7.11.1), and K13946 (AUX influx carrier). The Arabidopsis homologs of the eight candidate genes (*Glyma.02G211800, Glyma.16G122200, Glyma.17G178800, Glyma.07G134800, Glyma.20G222500, Glyma.07G147000, Glyma.16G129600*, and *Glyma.02G228200*) are associated with the plant growth pathway. National Center for Biotechnology Information (NCBI) indicated that these homologous genes mainly regulate important AUX response factors associated with the plant growth pathway, such as *AT4G23980*, which is annotated as an AUX response factor, and they may participate in the expression of negative regulators of AUX signaling in Arabidopsis (Kowalczuk, 1999; Cluis et al., 2004). AUX has an inhibitory effect on plant height, stem diameter, and root length (Zhang et al., 2018). Basnet et al. (1972) showed that the antiAUX triiodobenzoic acid reduced the node number subtending first pod. The AUX concentration could negatively regulate the pod setting of soybean (Nonokawa et al., 2007). Node and internode development are also regulated by AUX (Wilson et al., 1999). The stem elongation in some plant species is affected by AUX through the gibberellin, GA3-associated pathway (King et al., 2000; Leite et al., 2003; Walsh et al., 2006). Most of the genes identified in this study were involved in the AUX pathway; therefore, we propose that the soybean FPH is regulated by AUX. Additionally, *Glyma.02G211800, Glyma.07G134800, Glyma.07G147000, Glyma.16G129600*, and *Glyma.02G228200* are involved in the AUX pathway. While *Glyma.16G122200, Glyma.17G178800*, and *Glyma.20G222500* are not involved in the AUX pathway, they are involved in hormone network regulation and cell expansion. Thus, the FPH might be regulated by a complicated hormone regulatory pathway. Here, we identified the candidate genes underlying FPH. These high-expression genes may be involved in the FPH regulatory pathway, and further work is required to elucidate the molecular mechanisms of the candidate genes.

## Author Contributions

QC, XXW, ZQ, and DX designed the study. HJ, YYL, and HTQ analyzed the data and wrote the paper. YLL, HDQ, CDL, NW, RL, YZ, SH, JY, XYW, RZ, CYL, and ZH participated in correcting the manuscript. All authors have read and approved the final manuscript.

## Conflict of Interest Statement

The authors declare that the research was conducted in the absence of any commercial or financial relationships that could be construed as a potential conflict of interest.

## Footnotes

1 https://www.soybase.org/

2 https://phytozome.jgi.doe.gov/pz/portal.html#

3 http://www.invitrogen.com
