# Supplementary material for: Identification of Major QTLs Associated With First Pod Height and Candidate Gene Mining in Soybean
Source: Front Plant Sci. 2018 Sep 19;9:1280. doi: 10.3389/fpls.2018.01280 (PMC6157441; doi:10.3389/fpls.2018.01280)
Supplement: Supplementary file 1 [file Table_1.DOCX]

**Table S1** Database of gene prediction and QTL related information integration URL

| Databases | Uniform Resource Locator (URL) |
| --- | --- |
| Soybase | https://www.soybase.org/ |
| InterProScan | http://www.ebi.ac.uk/interpro/ |
| QuickGO | https://www.ebi.ac.uk/QuickGO/ |
| KEGG | http://www.kegg.jp/ |
| Phytozome | https://phytozome.jgi.doe.gov/pz/portal.html |
| NCBI | https://www.ncbi.nlm.nih.gov/ |
