# Supplementary material for: Identification of Major QTLs Associated With First Pod Height and Candidate Gene Mining in Soybean
Source: Front Plant Sci. 2018 Sep 19;9:1280. doi: 10.3389/fpls.2018.01280 (PMC6157441; doi:10.3389/fpls.2018.01280)
Supplement: Supplementary file 2 [file Table_2.DOCX]

| Table S2 Multiple Comparisons for FPH of 7 materials for qRT-PCR | | | | | | |
| --- | --- | --- | --- | --- | --- | --- |
| (I) FPH | (J) FPH |  | | | 95% Confidence Interval | |
|  |  | Mean Difference (I-J) | Std. Error | Mean Difference (I-J) | Std. Error | Mean Difference (I-J) |
| RIL-2 | RIL-86 | -6.99214^*^ | 2.50933 | .008 | -12.0462 | -1.9381 |
|  | RIL-101 | -9.27429^*^ | 2.59163 | .001 | -14.4941 | -4.0545 |
|  | RIL-112 | -1.03286 | 2.59163 | .692 | -6.2527 | 4.1869 |
|  | RIL-141 | -1.75714 | 2.59163 | .501 | -6.9769 | 3.4627 |
|  | Dongnong 594 | -3.37214 | 2.50933 | .186 | -8.4262 | 1.6819 |
|  | Charleston | -4.46714 | 2.50933 | .082 | -9.5212 | .5869 |
| RIL-86 | RIL-2 | 6.99214^*^ | 2.50933 | .008 | 1.9381 | 12.0462 |
|  | RIL-101 | -2.28214 | 2.50933 | .368 | -7.3362 | 2.7719 |
|  | RIL-112 | 5.95929^*^ | 2.50933 | .022 | .9052 | 11.0133 |
|  | RIL-141 | 5.23500^*^ | 2.50933 | .043 | .1809 | 10.2891 |
|  | Dongnong 594 | 3.62000 | 2.42425 | .142 | -1.2627 | 8.5027 |
|  | Charleston | 2.52500 | 2.42425 | .303 | -2.3577 | 7.4077 |
| RIL-101 | RIL-2 | 9.27429^*^ | 2.59163 | .001 | 4.0545 | 14.4941 |
|  | RIL-86 | 2.28214 | 2.50933 | .368 | -2.7719 | 7.3362 |
|  | RIL-112 | 8.24143^*^ | 2.59163 | .003 | 3.0216 | 13.4612 |
|  | RIL-141 | 7.51714^*^ | 2.59163 | .006 | 2.2973 | 12.7369 |
|  | Dongnong 594 | 5.90214^*^ | 2.50933 | .023 | .8481 | 10.9562 |
|  | Charleston | 4.80714 | 2.50933 | .062 | -.2469 | 9.8612 |
| RIL-112 | RIL-2 | 1.03286 | 2.59163 | .692 | -4.1869 | 6.2527 |
|  | RIL-86 | -5.95929^*^ | 2.50933 | .022 | -11.0133 | -.9052 |
|  | RIL-101 | -8.24143^*^ | 2.59163 | .003 | -13.4612 | -3.0216 |
|  | RIL-141 | -.72429 | 2.59163 | .781 | -5.9441 | 4.4955 |
|  | Dongnong 594 | -2.33929 | 2.50933 | .356 | -7.3933 | 2.7148 |
|  | Charleston | -3.43429 | 2.50933 | .178 | -8.4883 | 1.6198 |
| RIL-141 | RIL-2 | 1.75714 | 2.59163 | .501 | -3.4627 | 6.9769 |
|  | RIL-86 | -5.23500^*^ | 2.50933 | .043 | -10.2891 | -.1809 |
|  | RIL-101 | -7.51714^*^ | 2.59163 | .006 | -12.7369 | -2.2973 |
|  | RIL-112 | .72429 | 2.59163 | .781 | -4.4955 | 5.9441 |
|  | Dongnong 594 | -1.61500 | 2.50933 | .523 | -6.6691 | 3.4391 |
|  | Charleston | -2.71000 | 2.50933 | .286 | -7.7641 | 2.3441 |
| Dongnong 594 | RIL-2 | 3.37214 | 2.50933 | .186 | -1.6819 | 8.4262 |
|  | RIL-86 | -3.62000 | 2.42425 | .142 | -8.5027 | 1.2627 |
|  | RIL-101 | -5.90214^*^ | 2.50933 | .023 | -10.9562 | -.8481 |
|  | RIL-112 | 2.33929 | 2.50933 | .356 | -2.7148 | 7.3933 |
|  | RIL-141 | 1.61500 | 2.50933 | .523 | -3.4391 | 6.6691 |
|  | Charleston | -1.09500 | 2.42425 | .654 | -5.9777 | 3.7877 |
| Charleston | RIL-2 | 4.46714 | 2.50933 | .082 | -.5869 | 9.5212 |
|  | RIL-86 | -2.52500 | 2.42425 | .303 | -7.4077 | 2.3577 |
|  | RIL-101 | -4.80714 | 2.50933 | .062 | -9.8612 | .2469 |
|  | RIL-112 | 3.43429 | 2.50933 | .178 | -1.6198 | 8.4883 |
|  | RIL-141 | 2.71000 | 2.50933 | .286 | -2.3441 | 7.7641 |
|  | Dongnong 594 | 1.09500 | 2.42425 | .654 | -3.7877 | 5.9777 |
| *. The Mean Difference is significant at the 0.05 level. | | | | | | |
