# Supplementary material for: Identification of Major QTLs Associated With First Pod Height and Candidate Gene Mining in Soybean
Source: Front Plant Sci. 2018 Sep 19;9:1280. doi: 10.3389/fpls.2018.01280 (PMC6157441; doi:10.3389/fpls.2018.01280)
Supplement: Supplementary file 3 [file Table_3.DOCX]

**Table S3** The primer sequences for candidate genes for RT-qPCR

| Gene_id | Primer sequence (5ꞌ - 3ꞌ) | | Annealing tempreture | Extension time |
| --- | --- | --- | --- | --- |
| *Glyma.07G134800* | F | CCTCTCATAAATTCTCTGTCAACGC | 60℃ | 1 min |
|  | R | TGACCAATGAATCTAAATCCGTGAC |  |  |
| *Glyma.02G211800* | F | AGAGGCATTTGATTTGGATA | 60℃ | 1 min |
|  | R | GAGATGGGAGAAGAAGACGAG |  |  |
| *Glyma.16G122200* | F | CGTCACATAACCGTTCCTCTG | 60℃ | 1 min |
|  | R | CCCACAAACCCTTCCTCACC |  |  |
| *Glyma.02G228200* | F | CAACCTTGGTGGTGCCTGATG | 60℃ | 1 min |
|  | R | TGTTAATATCCCTCCCCTACGCG |  |  |
| *Glyma.20G222500* | F | CAAAATCATCAATCCCTCTTCTCAA | 60℃ | 1 min |
|  | R | GGAAGGGAATCGTTTGAGGGT |  |  |
| *Glyma.17G178800* | F | CAATACACGCCATTTTTCATTTCTC | 60℃ | 1 min |
|  | R | GAAACAAAACAACAACGGCAATAACT |  |  |
| *Glyma.07G147000* | F | TACAAATCACAAACACAAAGTCTCAT | 60℃ | 1 min |
|  | R | GTTGGAATGCGTGGACAAAACAGAA |  |  |
| *GmUKN1* | F | TGGT GCTG CCGC TATT TACT G | 60℃ | 1 min |
|  | R | GGTG GAAG GAAC TGCT AACA ATC |  |  |
