# Supplementary material for: Identification of Major QTLs Associated With First Pod Height and Candidate Gene Mining in Soybean
Source: Front Plant Sci. 2018 Sep 19;9:1280. doi: 10.3389/fpls.2018.01280 (PMC6157441; doi:10.3389/fpls.2018.01280)
Supplement: Supplementary file 4 [file Table_4.DOCX]

| Precipitation from 2006 to 2015 in Harbin (mm) | | | | | | | | | | | | | |
| --- | --- | --- | --- | --- | --- | --- | --- | --- | --- | --- | --- | --- | --- |
|  | January | February | March | April | May | June | July | August | September | October | November | December | Annual average |
| 2006 | 6.4 | 0.8 | 4.0 | 8.8 | 8.6 | 141.8 | 169.5 | 35.0 | 68.0 | 31.7 | 12.3 | 1.0 | 487.9 |
| 2007 | 7.3 | 16.0 | 40.2 | 12.4 | 87.9 | 74.9 | 68.7 | 53.2 | 62.7 | 9.5 | 2.7 | 8.6 | 444.1 |
| 2008 | 0.0 | 0.0 | 21.8 | 31.3 | 71.3 | 57.4 | 94.8 | 46.1 | 80.4 | 18.0 | 9.3 | 8.6 | 439.0 |
| 2009 | 6.8 | 4.0 | 25.8 | 34.4 | 28.0 | 178.7 | 106.1 | 66.9 | 37.2 | 24.4 | 10.4 | 11.4 | 534.1 |
| 2010 | 4.6 | 17.6 | 26.8 | 44.4 | 115.6 | 68.3 | 184.7 | 57.8 | 16.5 | 13.9 | 22.2 | 18.9 | 591.3 |
| 2013 | 2.1 | 16.5 | 8.8 | 10.8 | 73.5 | 86.4 | 198.0 | 125.7 | 31.5 | 58.2 | 18.3 | 3.7 | 633.5 |
| 2014 | 0.8 | 1.2 | 1.3 | 6.1 | 91.4 | 56.8 | 115.5 | 83.8 | 32.2 | 14.1 | 1.2 | 11.4 | 415.8 |
| 2015 | 0.8 | 14.1 | 2.5 | 6.6 | 77.6 | 77.3 | 52.9 | 110.5 | 24.8 | 30.0 | 5.5 | 17.5 | 420.1 |

Table S4 Precipitation from 2006 to 2015 in Harbin
