# Supplementary material for: Identification of Major QTLs Associated With First Pod Height and Candidate Gene Mining in Soybean
Source: Front Plant Sci. 2018 Sep 19;9:1280. doi: 10.3389/fpls.2018.01280 (PMC6157441; doi:10.3389/fpls.2018.01280)
Supplement: Supplementary file 5 [file Table_5.DOCX]

Table S5 Mean temperature from 2006 to 2015 in Harbin

| Mean temperature from 2006 to 2015 in Harbin (℃) | | | | | | | | | | | | | |
| --- | --- | --- | --- | --- | --- | --- | --- | --- | --- | --- | --- | --- | --- |
|  | January | February | March | April | May | June | July | August | September | October | November | December | Annual average |
| 2006 | -17.7 | -12.7 | -2.8 | 5.9 | 17.1 | 19.9 | 23.4 | 23.1 | 16.2 | 7.4 | -4.5 | -12.1 | 5.3 |
| 2007 | -11.5 | -7.0 | -3.3 | 7.1 | 14.9 | 23.7 | 23.3 | 22.7 | 16.7 | 7.5 | -3.8 | -10.6 | 6.6 |
| 2008 | -17.6 | -10.5 | 2.7 | 11.3 | 14.4 | 22.5 | 24.4 | 23.2 | 16.4 | 8.4 | -4.4 | -11.6 | 6.6 |
| 2009 | -16.0 | -11.5 | -3.9 | 9.7 | 18.4 | 18.9 | 22.6 | 22.2 | 15.6 | 7.1 | -7.0 | -16.7 | 5.0 |
| 2010 | -17.1 | -15.5 | -7.2 | 4.0 | 16.0 | 25.5 | 23.4 | 22.0 | 16.5 | 6.4 | -3.6 | -16.3 | 4.5 |
| 2013 | -21.1 | -16.4 | -7.4 | 4.4 | 17.9 | 21.4 | 23.9 | 22.5 | 15.8 | 7.0 | -2.6 | -14.0 | 4.3 |
| 2014 | -18.3 | -15.5 | -1.0 | 10.3 | 14.3 | 22.9 | 23.1 | 21.9 | 15.5 | 6.4 | -1.9 | -16.9 | 5.1 |
| 2015 | -15.8 | -11.3 | -1.3 | 8.6 | 14.2 | 22.1 | 23.6 | 22.8 | 16.2 | 7.2 | -4.9 | -14.0 | 5.6 |
