# Supplementary material for: Identification of Major QTLs Associated With First Pod Height and Candidate Gene Mining in Soybean
Source: Front Plant Sci. 2018 Sep 19;9:1280. doi: 10.3389/fpls.2018.01280 (PMC6157441; doi:10.3389/fpls.2018.01280)
Supplement: Supplementary file 6 [file Table_6.DOCX]

Table S6 Sunshine hour from 2006 to 2015 in Harbin

| Sunshine hour from 2006 to 2015 in Harbin (h) | | | | | | | | | | | | | |
| --- | --- | --- | --- | --- | --- | --- | --- | --- | --- | --- | --- | --- | --- |
|  | January | February | March | April | May | June | July | August | September | October | November | December | Annual |
| 2006 | 111.4 | 153.8 | 180.7 | 176.7 | 261.7 | 192.7 | 215.2 | 222.4 | 242.7 | 213.2 | 141.4 | 116.0 | 2227.9 |
| 2007 | 117.0 | 173.5 | 184.8 | 226.6 | 178.1 | 270.0 | 283.6 | 236.0 | 205.8 | 232.4 | 181.8 | 89.6 | 2379.2 |
| 2008 | 177.7 | 184.6 | 198.7 | 203.8 | 230.6 | 228.0 | 196.6 | 234.5 | 250.7 | 177.8 | 132.7 | 122.1 | 2337.8 |
| 2009 | 98.3 | 147.0 | 207.1 | 220.9 | 277.4 | 173.6 | 210.2 | 237.3 | 221.0 | 196.6 | 136.4 | 83.1 | 2208.9 |
| 2010 | 105.0 | 157.0 | 177.1 | 195.1 | 198.1 | 287.4 | 178.7 | 203.1 | 264.5 | 176.8 | 133.4 | 75.9 | 2152.1 |
| 2013 | 94.1 | 166.5 | 218.9 | 202.3 | 240.7 | 151.7 | 195.1 | 163.7 | 230.1 | 158.5 | 106.2 | 95.7 | 2023.5 |
| 2014 | 129.6 | 164.1 | 228.7 | 267.0 | 127.5 | 216.8 | 159.9 | 208.1 | 184.4 | 120.4 | 149.5 | 99.9 | 2055.9 |
| 2015 | 142.1 | 134.8 | 209.6 | 191.0 | 156.9 | 226.7 | 262.9 | 152.8 | 209.2 | 178.3 | 111.1 | 115.3 | 2090.7 |
