# Supplementary material for: Identification of Major QTLs Associated With First Pod Height and Candidate Gene Mining in Soybean
Source: Front Plant Sci. 2018 Sep 19;9:1280. doi: 10.3389/fpls.2018.01280 (PMC6157441; doi:10.3389/fpls.2018.01280)
Supplement: Supplementary file 7 [file Table_7.DOCX]

Table S7 Multiple Comparisons for FPH across 8 years

| (I)Year | (J) Year |  | | | 95% Confidence Interval | |
| --- | --- | --- | --- | --- | --- | --- |
|  |  | Mean Difference (I-J) | Std. Error | Sig. | lower Bound | Upper Bound |
| 2006 | 2007 | .07818 | .45988 | .865 | -.8241 | .9805 |
|  | 2008 | -10.92874* | .45988 | .000 | -11.8310 | -10.0264 |
|  | 2009 | -3.62371* | .46066 | .000 | -4.5275 | -2.7199 |
|  | 2010 | .35646 | .45988 | .438 | -.5458 | 1.2588 |
|  | 2013 | 6.19350* | .47272 | .000 | 5.2660 | 7.1210 |
|  | 2014 | 1.92164* | .45988 | .000 | 1.0194 | 2.8239 |
|  | 2015 | 1.53797* | .45988 | .001 | .6357 | 2.4403 |
| 2007 | 2006 | -.07818 | .45988 | .865 | -.9805 | .8241 |
|  | 2008 | -11.00691* | .45909 | .000 | -11.9077 | -10.1062 |
|  | 2009 | -3.70188* | .45988 | .000 | -4.6042 | -2.7996 |
|  | 2010 | .27829 | .45909 | .545 | -.6225 | 1.1790 |
|  | 2013 | 6.11532* | .47195 | .000 | 5.1893 | 7.0413 |
|  | 2014 | 1.84347* | .45909 | .000 | .9427 | 2.7442 |
|  | 2015 | 1.45980* | .45909 | .002 | .5590 | 2.3605 |
| 2008 | 2006 | 10.92874* | .45988 | .000 | 10.0264 | 11.8310 |
|  | 2007 | 11.00691* | .45909 | .000 | 10.1062 | 11.9077 |
|  | 2009 | 7.30503* | .45988 | .000 | 6.4027 | 8.2073 |
|  | 2010 | 11.28520* | .45909 | .000 | 10.3845 | 12.1860 |
|  | 2013 | 17.12224* | .47195 | .000 | 16.1963 | 18.0482 |
|  | 2014 | 12.85038* | .45909 | .000 | 11.9496 | 13.7511 |
|  | 2015 | 12.46671* | .45909 | .000 | 11.5660 | 13.3675 |
| 2009 | 2006 | 3.62371* | .46066 | .000 | 2.7199 | 4.5275 |
|  | 2007 | 3.70188* | .45988 | .000 | 2.7996 | 4.6042 |
|  | 2008 | -7.30503* | .45988 | .000 | -8.2073 | -6.4027 |
|  | 2010 | 3.98017* | .45988 | .000 | 3.0779 | 4.8825 |
|  | 2013 | 9.81721* | .47272 | .000 | 8.8897 | 10.7447 |
|  | 2014 | 5.54535* | .45988 | .000 | 4.6431 | 6.4476 |
|  | 2015 | 5.16168* | .45988 | .000 | 4.2594 | 6.0640 |
| 2010 | 2006 | -.35646 | .45988 | .438 | -1.2588 | .5458 |
|  | 2007 | -.27829 | .45909 | .545 | -1.1790 | .6225 |
|  | 2008 | -11.28520* | .45909 | .000 | -12.1860 | -10.3845 |
|  | 2009 | -3.98017* | .45988 | .000 | -4.8825 | -3.0779 |
|  | 2013 | 5.83703* | .47195 | .000 | 4.9111 | 6.7630 |
|  | 2014 | 1.56518* | .45909 | .001 | .6644 | 2.4659 |
|  | 2015 | 1.18151* | .45909 | .010 | .2808 | 2.0823 |
| 2013 | 2006 | -6.19350* | .47272 | .000 | -7.1210 | -5.2660 |
|  | 2007 | -6.11532* | .47195 | .000 | -7.0413 | -5.1893 |
|  | 2008 | -17.12224* | .47195 | .000 | -18.0482 | -16.1963 |
|  | 2009 | -9.81721* | .47272 | .000 | -10.7447 | -8.8897 |
|  | 2010 | -5.83703* | .47195 | .000 | -6.7630 | -4.9111 |
|  | 2014 | -4.27185* | .47195 | .000 | -5.1978 | -3.3459 |
|  | 2015 | -4.65553* | .47195 | .000 | -5.5815 | -3.7295 |
| 2014 | 2006 | -1.92164* | .45988 | .000 | -2.8239 | -1.0194 |
|  | 2007 | -1.84347* | .45909 | .000 | -2.7442 | -.9427 |
|  | 2008 | -12.85038* | .45909 | .000 | -13.7511 | -11.9496 |
|  | 2009 | -5.54535* | .45988 | .000 | -6.4476 | -4.6431 |
|  | 2010 | -1.56518* | .45909 | .001 | -2.4659 | -.6644 |
|  | 2013 | 4.27185* | .47195 | .000 | 3.3459 | 5.1978 |
|  | 2015 | -.38367 | .45909 | .403 | -1.2844 | .5171 |
| 2015 . | 2006 | -1.53797* | .45988 | .001 | -2.4403 | -.6357 |
|  | 2007 | -1.45980* | .45909 | .002 | -2.3605 | -.5590 |
|  | 2008 | -12.46671* | .45909 | .000 | -13.3675 | -11.5660 |
|  | 2009 | -5.16168* | .45988 | .000 | -6.0640 | -4.2594 |
|  | 2010 | -1.18151* | .45909 | .010 | -2.0823 | -.2808 |
|  | 2013 | 4.65553* | .47195 | .000 | 3.7295 | 5.5815 |
|  | 2014 | .38367 | .45909 | .403 | -.5171 | 1.2844 |

*. The Mean Difference is significant at the 0.05 level.
