# Supplementary material for: Identification of Major QTLs Associated With First Pod Height and Candidate Gene Mining in Soybean
Source: Front Plant Sci. 2018 Sep 19;9:1280. doi: 10.3389/fpls.2018.01280 (PMC6157441; doi:10.3389/fpls.2018.01280)
Supplement: Supplementary file 8 [file Table_8.DOCX]

**Table S8** Epistatic QTL interaction analyze of the FPH in RIL

| Trait Name | QTL | Marker Interval | QTL Position | QTL | Marker Interval | QTL Position | LOD | PVE(%) | Add1 | Add2 | Add by Add |
| --- | --- | --- | --- | --- | --- | --- | --- | --- | --- | --- | --- |
| 2006HRB | qPH-d1a-2 | Mark659759  Mark590954 | 40 | qPH-d1a-7 | Mark586999  Mark588867 | 70 | 8.62 | 1.58 | -6.91 | 6.86 | -7.30 |
| 2006HRB | qPH-d1a-2 | Mark659759  Mark590954 | 40 | qPH-c1-1 | Mark825067  Mark775719 | 5 | 9.26 | 1.63 | -7.14 | 6.59 | -7.58 |
| 2006HRB | qPH-d1b-5 | Mark1044248  Mark984446 | 110 | qPH-f-4 | Mark103009  Mark101936 | 75 | 11.18 | 1.59 | 9.34 | -10.37 | -9.71 |
| 2006HRB | qPH-d1b-7 | Mark993815  Mark1026635 | 135 | qPH-b2-3 | Mark715047  Mark684376 | 40 | 9.35 | 1.51 | 9.32 | -9.83 | -10.06 |
| 2006HRB | qPH-d1b-7 | Mark993815Mark1026635 | 135 | qPH-i-5 | Mark1152218  Mark1153946 | 70 | 13.57 | 1.56 | 9.43 | -9.63 | -10.27 |
| 2006HRB | qPH-n-3 | Mark887627  Mark860580 | 20 | qPH-n-4 | Mark844000  Mark889539 | 25 | 9.59 | 1.59 | 9.28 | -10.14 | -9.69 |
| 2006HRB | qPH-c1-4 | Mark815895  Mark766568 | 70 | qPH-c2-3 | Mark467521  Mark502744 | 80 | 9.18 | 1.61 | 8.92 | -9.68 | -10.60 |
| 2006HRB | *qPH-c1-1* | Mark825067  Mark775719 | 5 | *qPH-f-4* | Mark103009  Mark101936 | 75 | 9.02 | 1.64 | 6.61 | -7.64 | -7.09 |
| Trait Name | QTL | Marker Interval | QTL Position | QTL | Marker Interval | QTL Position | LOD | PVE(%) | Add1 | Add2 | Add by Add |
| 2006HRB | *qPH-c1-1* | Mark825067  Mark775719 | 5 | *qPH-e-4* | Mark733  Mark37514 | 140 | 5.29 | 1.21 | 4.13 | -5.13 | -5.10 |
| 2006HRB | *qPH-c1-1* | Mark825067  Mark775719 | 5 | *qPH-l-8* | Mark93812  1Mark956316 | 185 | 6.43 | 1.25 | 4.46 | -5.35 | -5.51 |
| 2006HRB | *qPH-a1-1* | Mark336245  Mark314896 | 10 | *qPH-a1-3* | Mark356416  Mark299635 | 20 | 9.14 | 1.50 | -9.63 | 9.53 | -9.87 |
| 2006HRB | *qPH-m-1* | Mark582982  Mark532996 | 0 | *qPH-m-3* | Mark563339  Mark558816 | 40 | 6.49 | 1.23 | 5.56 | -6.56 | -6.50 |
| 2006HRB | *qPH-m-1* | Mark582982  Mark532996 | 0 | *qPH-a2-5* | Mark1341757  Mark1365346 | 65 | 5.30 | 1.08 | 3.78 | -4.85 | -5.34 |
| 2006HRB | *qPH-m-1* | Mark582982  Mark532996 | 0 | *qPH-k-3* | Mark255546  Mark280462 | 25 | 5.10 | 1.24 | 5.57 | -6.68 | -6.38 |
| 2006HRB | *qPH-m-1* | Mark582982  Mark532996 | 0 | *qPH-f-1* | Mark110983  Mark105927 | 5 | 6.81 | 1.26 | 5.56 | -6.77 | -6.29 |
| 2006HRB | *qPH-m-4* | Mark553961  Mark558964 | 45 | *qPH-e-2* | Mark4453  Mark28176 | 130 | 6.61 | 1.65 | -6.85 | 5.99 | -7.57 |
| 2006HRB | *qPH-m-1* | Mark582982  Mark532996 | 0 | *qPH-d2-1* | Mark1395834  Mark1428459 | 15 | 5.45 | 1.07 | 3.80 | -4.87 | -5.31 |
| Trait Name | QTL | Marker Interval | QTL Position | QTL | Marker Interval | QTL Position | LOD | PVE(%) | Add1 | Add2 | Add by Add |
| 2006HRB | *qPH-k-4* | Mark263670  Mark261508 | 35 | *qPH-k-5* | Mark248126  Mark268890 | 40 | 10.59 | 1.59 | -7.22 | 6.63 | -7.18 |
| 2006HRB | *qPH-o-1* | Mark430156  Mark407726 | 30 | *qPH-o-2* | Mark371795  Mark393942 | 60 | 8.14 | 1.51 | 9.84 | -9.46 | -9.65 |
| 2006HRB | *qPH-h-4* | Mark1097732  Mark1082022 | 45 | *qPH-h-5* | Mark1106927  Mark1064245 | 50 | 8.47 | 1.58 | -9.40 | 8.98 | -10.35 |
| 2006HRB | *qPH-h-3* | Mark1117004  Mark1060032 | 35 | *qPH-e-1* | Mark76239  Mark47376 | 125 | 10.26 | 1.60 | -10.20 | 9.11 | -9.87 |
| 2006HRB | *qPH-f-4* | Mark103009  Mark101936 | 75 | *qPH-f-5* | Mark101936  Mark123391 | 80 | 11.30 | 1.60 | -10.44 | 9.64 | -9.38 |
| 2006HRB | *qPH-f-1* | Mark110983  Mark105927 | 5 | *qPH-e-1* | Mark76239  Mark47376 | 125 | 7.55 | 1.61 | -10.20 | 9.12 | -9.89 |
| 2006HRB | *qPH-b2-1* | Mark685699  Mark689597 | 5 | *qPH-b2-2* | Mark703197  Mark678611 | 20 | 8.60 | 1.53 | 9.14 | -9.19 | -10.29 |
| 2006HRB | *qPH-e-1* | Mark76239  Mark47376 | 125 | *qPH-e-2* | Mark4453  Mark28176 | 130 | 11.03 | 1.59 | 9.14 | -10.08 | -9.98 |
| 2006HRB | *qPH-j-5* | Mark1229446  Mark1201956 | 75 | *qPH-j-6* | Mark1240744  Mark1227663 | 80 | 9.50 | 1.55 | -9.12 | 9.54 | -10.12 |
| Trait Name | QTL | Marker Interval | QTL Position | QTL | Marker Interval | QTL Position | LOD | PVE(%) | Add1 | Add2 | Add by Add |
| 2006HRB | *qPH-d2-6* | Mark1385152  Mark1394819 | 120 | *qPH-d2-7* | Mark1431677  Mark1382419 | 125 | 10.33 | 1.56 | -9.32 | 10.04 | -9.65 |
| 2006HRB | *qPH-d2-3* | Mark1430758  Mark1430459 | 70 | *qPH-g-3* | Mark161603  Mark139879 | 75 | 9.87 | 1.62 | -6.96 | 6.68 | -7.52 |
| 2006HRB | *qPH-g-1* | Mark208499  Mark142054 | 25 | *qPH-g-2* | Mark188906  Mark205529 | 35 | 9.70 | 1.51 | 9.88 | -9.51 | -9.68 |
| 2006HRB | *qPH-g-5* | Mark140447  Mark182578 | 145 | *qPH-i-5* | Mark1152218  Mark1153946 | 70 | 5.15 | 0.73 | 3.92 | -3.42 | -4.05 |
| 2006HRB | *qPH-l-2* | Mark967921  Mark958955 | 15 | *qPH-l-3* | Mark953249  Mark958292 | 25 | 9.60 | 1.54 | -6.55 | 6.67 | -7.26 |
| 2006HRB | *qPH-i-2* | Mark1147619  Mark1155167 | 35 | *qPH-i-5* | Mark1152218  Mark1153946 | 70 | 13.35 | 1.54 | 9.65 | -9.42 | -10.05 |
| 2007HRB | *qPH-d1b-6* | Mark1054121  Mark1049036 | 120 | *qPH-k-6* | Mark298039  Mark257702 | 80 | 6.07 | 9.49 | 4.93 | -4.39 | -4.84 |
| 2007HRB | *qPH-l-4* | Mark957172  Mark967577 | 55 | *qPH-l-5* | Mark972700  Mark915588 | 60 | 5.17 | 11.93 | 4.16 | -3.73 | -5.82 |
| 2008HRB | *qPH-c1-3* | Mark779987  Mark773285 | 55 | *qPH-d2-2* | Mark1402080  Mark1422077 | 40 | 5.01 | 16.66 | 1.40 | -1.64 | -2.86 |
| Trait Name | QTL | Marker Interval | QTL Position | QTL | Marker Interval | QTL Position | LOD | PVE(%) | Add1 | Add2 | Add by Add |
| 2009HRB | *qPH-d1a-5* | Mark627649  Mark589174 | 55 | *qPH-c1-2* | Mark767412  Mark823792 | 10 | 12.34 | 1.30 | -6.19 | 6.25 | -6.50 |
| 2009HRB | *qPH-d1a-1* | Mark660242  Mark612085 | 30 | *qPH-a2-1* | Mark1312817  Mark1334259 | 30 | 8.81 | 1.33 | -5.93 | 6.54 | -6.51 |
| 2009HRB | *qPH-d1a-4* | Mark594624  Mark626799 | 50 | *qPH-h-6* | Mark1109889  Mark1111552 | 60 | 10.48 | 1.30 | -6.20 | 6.32 | -6.40 |
| 2009HRB | *qPH-d1a-4* | Mark594624  Mark626799 | 50 | *qPH-g-3* | Mark161603  Mark139879 | 75 | 11.47 | 1.32 | -6.31 | 6.07 | -6.67 |
| 2009HRB | *qPH-d1a-1* | Mark660242  Mark612085 | 30 | *qPH-i-1* | Mark1165824  Mark1165022 | 10 | 7.76 | 1.32 | -6.03 | 6.42 | -6.56 |
| 2009HRB | *qPH-d1b-2* | Mark1029765  Mark991902 | 55 | *qPH-d1b-3* | Mark1057042  Mark1024590 | 60 | 9.88 | 1.33 | 5.42 | -5.43 | -7.25 |
| 2009HRB | *qPH-d1b-7* | Mark993815  Mark1026635 | 135 | *qPH-k-5* | Mark248126  Mark268890 | 40 | 9.70 | 1.32 | 6.66 | -6.30 | -5.89 |
| 2009HRB | *qPH-d1b-8* | Mark1047449  Mark1022596 | 140 | *qPH-o-5* | Mark426603  Mark373880 | 95 | 6.79 | 1.31 | 6.03 | -6.05 | -6.65 |
| 2009HRB | *qPH-d1b-7* | Mark993815  Mark1026635 | 135 | *qPH-h-1* | Mark1093655  Mark1088962 | 25 | 6.47 | 1.30 | 6.56 | -6.13 | -6.07 |
| Trait Name | QTL | Marker Interval | QTL Position | QTL | Marker Interval | QTL Position | LOD | PVE(%) | Add1 | Add2 | Add by Add |
| 2009HRB | *qPH-d1b-4* | Mark1039314  Mark1037999 | 85 | *qPH-f-8* | Mark105385  Mark103240 | 135 | 7.60 | 1.30 | -6.16 | 6.46 | -6.25 |
| 2009HRB | *qPH-d1b-7* | Mark993815  Mark1026635 | 135 | *qPH-b2-1* | Mark685699  Mark689597 | 5 | 9.69 | 1.32 | 6.67 | -6.28 | -5.88 |
| 2009HRB | *qPH-d1b-7* | Mark993815  Mark1026635 | 135 | *qPH-g-4* | Mark228699  Mark194361 | 90 | 8.87 | 0.89 | 7.48 | -6.99 | -7.22 |
| 2009HRB | *qPH-d1b-6* | Mark1054121  Mark1049036 | 120 | *qPH-i-7* | Mark1142339  Mark1144932 | 130 | 6.23 | 0.78 | 4.58 | -4.96 | -5.30 |
| 2009HRB | *qPH-n-2* | Mark880629  Mark836626 | 15 | *qPH-n-4* | Mark844000  Mark889539 | 25 | 9.95 | 1.30 | 6.10 | -6.36 | -6.47 |
| 2009HRB | *qPH-n-4* | Mark844000  Mark889539 | 25 | *qPH-c1-2* | Mark767412  Mark823792 | 10 | 8.30 | 1.29 | -6.38 | 6.31 | -6.24 |
| 2009HRB | *qPH-n-4* | Mark844000  Mark889539 | 25 | *qPH-i-1* | Mark1165824  Mark1165022 | 10 | 6.20 | 1.30 | -6.38 | 6.37 | -6.17 |
| 2009HRB | *qPH-c1-4* | Mark815895  Mark766568 | 70 | *qPH-k-5* | Mark248126  Mark268890 | 40 | 5.10 | 1.26 | 5.20 | -5.14 | -4.92 |
| 2009HRB | *qPH-c1-2* | Mark767412  Mark823792 | 10 | *qPH-h-2* | Mark1092959  Mark1079508 | 30 | 12.14 | 1.30 | 6.28 | -6.23 | -6.45 |
| Trait Name | QTL | Marker Interval | QTL Position | QTL | Marker Interval | QTL Position | LOD | PVE(%) | Add1 | Add2 | Add by Add |
| 2009HRB | *qPH-c1-2* | Mark767412  Mark823792 | 10 | *qPH-d2-4* | Mark1433110  Mark1429797 | 105 | 7.26 | 1.33 | 6.23 | -6.62 | -6.09 |
| 2009HRB | *qPH-c1-2* | Mark767412  Mark823792 | 10 | *qPH-l-8* | Mark938121  Mark956316 | 185 | 5.86 | 1.30 | 6.24 | -6.14 | -6.49 |
| 2009HRB | *qPH-a1-1* | Mark336245  Mark314896 | 10 | *qPH-a1-2* | Mark314896  Mark300560 | 15 | 9.47 | 1.31 | -5.94 | 6.29 | -6.48 |
| 2009HRB | *qPH-c2-1* | Mark462584  Mark448518 | 50 | *qPH-c2-2* | Mark502497  Mark514227 | 65 | 7.13 | 1.32 | -6.75 | 6.76 | -5.81 |
| 2009HRB | *qPH-m-2* | Mark555767  Mark534251 | 35 | *qPH-m-3* | Mark563339  Mark558816 | 40 | 10.14 | 1.33 | 6.77 | -6.38 | -5.92 |
| 2009HRB | *qPH-m-4* | Mark553961  Mark558964 | 45 | *qPH-h-6* | Mark1109889  Mark1111552 | 60 | 5.51 | 0.61 | -2.13 | 1.87 | -2.51 |
| 2009HRB | *qPH-m-4* | Mark553961  Mark558964 | 45 | *qPH-e-2* | Mark4453  Mark28176 | 130 | 5.73 | 1.30 | -6.17 | 6.14 | -6.51 |
| 2009HRB | *qPH-a2-3* | Mark1364494  Mark1352508 | 40 | *qPH-a2-4* | Mark1315149  Mark1338805 | 45 | 10.99 | 1.32 | 6.25 | -5.91 | -6.51 |
| 2009HRB | *qPH-a2-2* | Mark1333038  Mark1339878 | 35 | *qPH-j-4* | Mark1222850  Mark1230923 | 55 | 5.09 | 0.63 | 2.59 | -2.52 | -2.58 |
| Trait Name | QTL | Marker Interval | QTL Position | QTL | Marker Interval | QTL Position | LOD | PVE(%) | Add1 | Add2 | Add by Add |
| 2009HRB | *qPH-b1-1* | Mark1297103  Mark1251867 | 10 | *qPH-b1-2* | Mark1288874  Mark1293478 | 15 | 8.03 | 1.30 | -6.09 | 6.30 | -6.42 |
| 2009HRB | *qPH-b1-2* | Mark1288874  Mark1293478 | 15 | *qPH-j-1* | Mark1244068  Mark1200474 | 25 | 5.66 | 1.30 | 6.43 | -6.24 | -6.23 |
| 2009HRB | *qPH-h-2* | Mark1092959  Mark1079508 | 30 | *qPH-h-6* | Mark1109889  Mark1111552 | 60 | 10.43 | 1.30 | -6.21 | 6.31 | -6.42 |
| 2009HRB | *qPH-h-2* | Mark1092959  Mark1079508 | 30 | *qPH-e-2* | Mark4453  Mark28176 | 130 | 6.14 | 1.30 | -6.18 | 6.23 | -6.49 |
| 2009HRB | *qPH-h-1* | Mark1093655  Mark1088962 | 25 | *qPH-d2-12* | Mark1393543  Mark1410805 | 185 | 6.22 | 1.29 | -6.28 | 6.39 | -6.25 |
| 2009HRB | *qPH-h-6* | Mark1109889  Mark1111552 | 60 | *qPH-l-1* | Mark931755  Mark932902 | 5 | 9.91 | 1.29 | 6.32 | -6.32 | -6.29 |
| 2009HRB | *qPH-f-6* | Mark109422  Mark135732 | 125 | *qPH-f-7* | Mark78800  Mark127196 | 130 | 10.47 | 1.32 | -6.77 | 6.72 | -5.84 |
| 2009HRB | *qPH-b2-4* | Mark687204  Mark730947 | 75 | *qPH-b2-5* | Mark715994  Mark714778 | 80 | 9.06 | 1.30 | 6.21 | -6.13 | -6.46 |
| 2009HRB | *qPH-e-2* | Mark4453  Mark28176 | 130 | *qPH-e-3* | Mark38609  Mark52903 | 135 | 12.57 | 1.33 | 5.79 | -5.88 | -6.85 |
| Trait Name | QTL | Marker Interval | QTL Position | QTL | Marker Interval | QTL Position | LOD | PVE(%) | Add1 | Add2 | Add by Add |
| 2009HRB | *qPH-j-2* | Mark1228752  Mark1196047 | 45 | *qPH-j-3* | Mark1215522  Mark1200764 | 50 | 8.87 | 1.30 | 6.38 | -6.13 | -6.35 |
| 2009HRB | *qPH-d2-8* | Mark1403766  Mark1417538 | 160 | *qPH-d2-11* | Mark1386640  Mark1423828 | 175 | 11.50 | 1.33 | -6.33 | 6.64 | -5.94 |
| 2009HRB | *qPH-d2-5* | Mark1395257  Mark1385960 | 110 | *qPH-g-3* | Mark161603  Mark139879 | 75 | 10.88 | 1.31 | -6.51 | 6.05 | -6.46 |
| 2009HRB | *qPH-g-6* | Mark223486  Mark203022 | 160 | *qPH-g-7* | Mark194270  Mark147187 | 165 | 8.45 | 1.26 | 5.13 | -5.07 | -5.10 |
| 2009HRB | *qPH-l-6* | Mark954320  Mark960524 | 75 | *qPH-l-7* | Mark927397  Mark956487 | 160 | 11.65 | 1.40 | 5.86 | -6.02 | -6.96 |
| 2009HRB | *qPH-i-2* | Mark1147619  Mark1155167 | 35 | *qPH-i-4* | Mark1124903  Mark1120723 | 45 | 8.30 | 1.29 | 6.31 | -6.25 | -6.34 |
| 2015HRB | *qPH-d1a-3* | Mark635607  Mark587057 | 45 | *qPH-d1a-6* | Mark598230  Mark669582 | 65 | 5.85 | 2.29 | -4.89 | 5.15 | -5.27 |
| 2015HRB | *qPH-d1b-1* | Mark1046851  Mark1003937 | 45 | *qPH-d1b-4* | Mark1039314  Mark1037999 | 85 | 5.01 | 2.46 | 5.47 | -4.88 | -5.02 |
| 2015HRB | *qPH-n-1* | Mark873841  Mark877388 | 10 | *qPH-n-3* | Mark887627  Mark860580 | 20 | 5.53 | 2.42 | 4.88 | -5.46 | -5.14 |
| Trait Name | QTL | Marker Interval | QTL Position | QTL | Marker Interval | QTL Position | LOD | PVE(%) | Add1 | Add2 | Add by Add |
| 2015HRB | *qPH-a1-1* | Mark336245  Mark314896 | 10 | *qPH-a1-2* | Mark314896  Mark300560 | 15 | 6.06 | 2.28 | -5.20 | 5.36 | -4.88 |
| 2015HRB | *qPH-k-1* | Mark230991  Mark252041 | 5 | *qPH-k-2* | Mark257245  Mark241572 | 10 | 6.12 | 2.28 | -5.19 | 4.90 | -5.24 |
| 2015HRB | *qPH-o-3* | Mark416941  Mark434892 | 85 | *qPH-o-4* | Mark415982  Mark404264 | 90 | 5.44 | 2.33 | -4.77 | 4.88 | -5.42 |
| 2015HRB | *qPH-h-4* | Mark1097732  Mark1082022 | 45 | *qPH-h-5* | Mark1106927  Mark1064245 | 50 | 5.62 | 2.11 | -3.21 | 3.24 | -3.75 |
| 2015HRB | *qPH-f-2* | Mark81695  Mark105636 | 35 | *qPH-f-3* | Mark128048  Mark117136 | 50 | 5.62 | 2.32 | -5.37 | 5.14 | -4.91 |
| 2015HRB | *qPH-b2-5* | Mark715994  Mark714778 | 80 | *qPH-b2-6* | Mark714778  Mark750587 | 85 | 6.76 | 2.26 | 5.21 | -4.97 | -5.10 |
| 2015HRB | *qPH-e-2* | Mark4453  Mark28176 | 130 | *qPH-e-3* | Mark38609  Mark52903 | 135 | 5.61 | 2.28 | 5.36 | -5.40 | -4.81 |
| 2015HRB | *qPH-d2-9* | Mark1426359  Mark1415292 | 165 | *qPH-d2-10* | Mark1381496  Mark1389100 | 170 | 5.88 | 2.47 | -5.20 | 5.63 | -4.68 |
| 2015HRB | *qPH-g-5* | Mark140447  Mark182578 | 145 | *qPH-i-6* | Mark1145543  Mark1152214 | 115 | 5.77 | 1.49 | 1.47 | -1.92 | -2.39 |
| Trait Name | QTL | Marker Interval | QTL Position | QTL | Marker Interval | QTL Position | LOD | PVE(%) | Add1 | Add2 | Add by Add |
| 2015HRB | *qPH-i-3* | Mark1138091  Mark1164065 | 40 | *qPH-i-4* | Mark1124903  Mark1120723 | 45 | 5.49 | 2.36 | 5.29 | -4.80 | -5.08 |
