# Supplementary material for: Identification of Major QTLs Associated With First Pod Height and Candidate Gene Mining in Soybean
Source: Front Plant Sci. 2018 Sep 19;9:1280. doi: 10.3389/fpls.2018.01280 (PMC6157441; doi:10.3389/fpls.2018.01280)
Supplement: Supplementary file 10 [file Table_10.DOCX]

**Table S10** Candidate genes involved in plant growth pathways

| Gm | Location(Mb) | Gene id | GO annotation | Homologe gene symbol | Homologe gene description | Reference |
| --- | --- | --- | --- | --- | --- | --- |
| Gm07 | 1.59-1.84 | Glyma.07G134800 | GO:0009725 | AT4G23980 | Arabidopsis thaliana auxin response factor 9 | ([Ulmasov et al. 1999](#_ENREF_6)) |
| Gm02 | 3.93-4.17 | Glyma.02G211800 |  | AT5G49980 | Auxin F-box protein 5 | ([Lee et al. 2013](#_ENREF_1)) |
| Gm16 | 0.25-3.17 | Glyma.16G122200 | GO:0006470,GO:0004722 | AT3G51370 | Protein phosphatase 2C family protein | ([Spartz et al. 2014](#_ENREF_5)) |
| Gm16 | 0.25-3.17 | Glyma.16G129600 | GO:0009733 | AT4G34760 | SAUR-like auxin-responsive protein family | (Mayer et al. 1999) |
| Gm02 | 3.93-4.17 | Glyma.02G228200 | GO:0003824,GO:0006470,GO:0004722 | AT2G30020 | Protein phosphatase 2C family protein mRNA | ([Shubchynskyy et al. 2017](#_ENREF_4)) |
| Gm20 | 4.56-4.57 | Glyma.20G222500 | GO:0016787 | AT2G39840 | Type one serine/threonine protein phosphatase 4 | ([Yue et al. 2016](#_ENREF_7)) |
| Gm17 | 2.66-2.78 | Glyma.17G178800 | GO:0006468,GO:0005524,GO:0004672 | AT5G66880 | Sucrose nonfermenting 1(SNF1)-related protein kinase 2.3 | ([Li et al. 2000](#_ENREF_2)) |
| Gm07 | 1.59-1.84 | Glyma.07G147000 |  | AT2G38120 | Transmembrane amino acid transporter family protein | ([Li et al. 2015](#_ENREF_3)) |
